# The age distribution of mortality due to influenza: pandemic and peri-pandemic

**DOI:** 10.1186/1741-7015-10-162

**Published:** 2012-12-12

**Authors:** Tom Reichert, Gerardo Chowell, Jonathan A McCullers

**Affiliations:** 1 Entropy Research Institute, 345 S. Great Road, Lincoln, 01773, Massachusetts, USA; 2 Mathematical, Computational and Modeling Sciences Center, School of Human Evolution and Social Change, Arizona State University, Tempe, 85287, Arizona, USA; 3 Division of Epidemiology and Population Studies, Fogarty International Center, National Institutes of Health, 31 Center Dr-MSC 2220, Bethesda, 20892-2220, Maryland, USA; 4 Department of Infectious Diseases, St. Jude Children's Research Hospital, 262 Danny Thomas Place, Memphis, 38104, Tennessee, USA; 5 Department of Pediatrics, University of Tennessee Health Sciences Center, 50 N. Dunlap, Memphis, 38104, Tennessee, USA

**Keywords:** Pandemic influenza, mortality due to influenza, recycling, pandemic attack rates, vaccination, protective immunity

## Abstract

**Background:**

Pandemic influenza is said to 'shift mortality' to younger age groups; but also to spare a subpopulation of the elderly population. Does one of these effects dominate? Might this have important ramifications?

**Methods:**

We estimated age-specific excess mortality rates for all-years for which data were available in the 20th century for Australia, Canada, France, Japan, the UK, and the USA for people older than 44 years of age. We modeled variation with age, and standardized estimates to allow direct comparison across age groups and countries. Attack rate data for four pandemics were assembled.

**Results:**

For nearly all seasons, an exponential model characterized mortality data extremely well. For seasons of emergence and a variable number of seasons following, however, a subpopulation above a threshold age invariably enjoyed reduced mortality. 'Immune escape', a stepwise increase in mortality among the oldest elderly, was observed a number of seasons after both the A(H2N2) and A(H3N2) pandemics. The number of seasons from emergence to escape varied by country. For the latter pandemic, mortality rates in four countries increased for younger age groups but only in the season following that of emergence. Adaptation to both emergent viruses was apparent as a progressive decrease in mortality rates, which, with two exceptions, was seen only in younger age groups. Pandemic attack rate variation with age was estimated to be similar across four pandemics with very different mortality impact.

**Conclusions:**

In all influenza pandemics of the 20th century, emergent viruses resembled those that had circulated previously within the lifespan of then-living people. Such individuals were relatively immune to the emergent strain, but this immunity waned with mutation of the emergent virus. An immune subpopulation complicates and may invalidate vaccine trials. Pandemic influenza does not 'shift' mortality to younger age groups; rather, the mortality level is reset by the virulence of the emerging virus and is moderated by immunity of past experience. In this study, we found that after immune escape, older age groups showed no further mortality reduction, despite their being the principal target of conventional influenza vaccines. Vaccines incorporating variants of pandemic viruses seem to provide little benefit to those previously immune. If attack rates truly are similar across pandemics, it must be the case that immunity to the pandemic virus does not prevent infection, but only mitigates the consequences.

## Background

**Figure 1:**
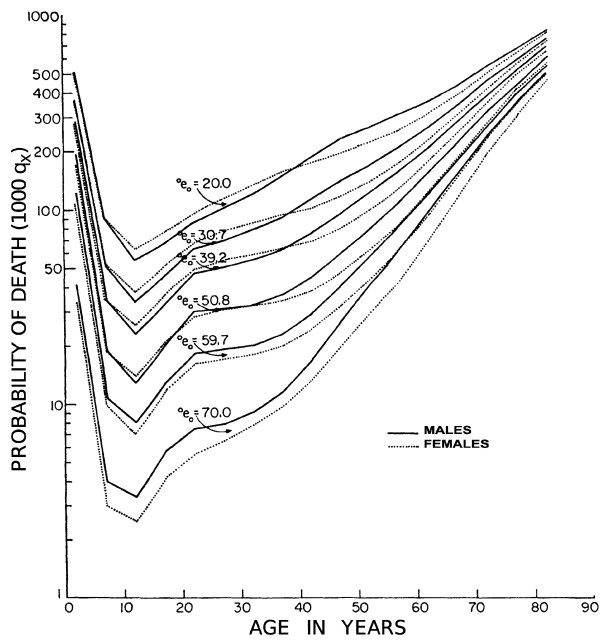
**The probability of death versus age for human populations of successively longer median life expectancy (expressed as deaths per 1000 population)**. Source: Department of Social Affairs. Population Branch, Age and Sex Patterns of Mortality: Model Life Tables for Underdeveloped Countries. *Population Studies*, No. 22, New York, United Nations, 1955.

**Figure 2:**
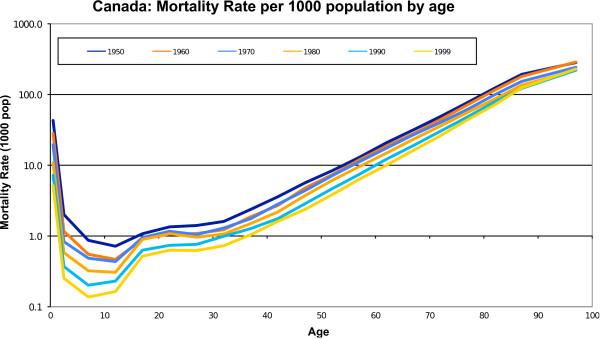
**The evolution of observed age-specific all-cause mortality rates, scaled per 1000 population, in Canada over the second half of the 20th century**. This evolution dovetails with the historical model of socioeconomic evolution.

Viewed against the backdrop of social evolution, the age distribution of the probability of death in human populations has a checkmark-like shape. The top curve in Figure 1 characterizes a society with life expectancy at birth of 20 years, about that for ancient Greece at the time of Pericles (448 to 404 BC). The lowest curve depicts the deathscape of a modern economically developed country, around 1950. The nested checkmark shapes derive from the fact that the mortality of infants and young children has always been greater than that of 8- to 12-year-olds. Above this age range, mortality rates steadily increase. The most important additional feature is that the segment of each curve for ages of around 40 years and older approximates a straight line Because the ordinate of the graph is plotted on a logarithmic scale, a straight line indicates that human mortality increases about exponentially with age from young middle age onwards. Over the approximately 2,400 years between 425 BC and 1950 AD, the death rate of 80-year-olds decreased by approximately 50%, or approximately 2% per century. Over the next 50 years, this death rate fell by an additional one-third in economically developed countries such as Canada (Figure 2). This is perhaps less remarkable than the fall of 70 to 80% in mortality for children from infancy to 12 years old over the same fifty years, but is evidence of true progress in human life extension.

**Figure 3:**
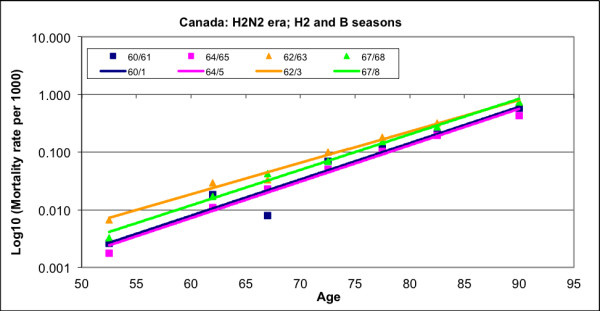
**A semi-log plot of excess all-cause mortality variation with age for epidemic seasons during the era of circulation of influenza A(H2N2) viruses in Canada**. The 1960/61 and 1964/5 seasons were dominated by B-type viruses, and the 1962/3 and 1967/8 seasons were dominated in mortality by H2N2 viruses.

Influenza-attributable or excess mortality has also been found to be exponential with age [1,2]. Figure 3 displays excess all-cause (AC) mortality rates (estimated using the technique of this paper) for Canada, for a selected set of epidemic seasons in the era of circulation of A(H2N2) viruses in seasons dominated by H2 and B viruses. Trend lines are plotted for these seasons. It is clear that an exponential relationship exists, and that this relationship is quite similar for seasons of a type. Excess AC mortality rates in 85- to 90-year-olds are about 250 times greater than those for 45 to 50-year-olds, whereas AC total mortality rates for 85 to 90-year-olds are only about 75 times larger than the rates for 45- to 50-year- olds. A characteristic of exponential processes is that a proportional change in the process occurs as a result of a fixed change in the driver of the process, here, age. The age increase associated with a doubling of the AC mortality rate was 7.15 years in Canada in 1999, while the doubling age increment for influenza-attributable mortality was 5.33 years. The exponential variation with age is, therefore, steeper for influenza-attributable mortality than for AC mortality. Seasonal influenza, as a mortal force, is of unusual consequence to the increasingly elderly population.

Two generalizations can be made from Figure 3. First, it is clear that excess mortality was higher in seasons in which mortality was dominated by influenza A-type viruses. The pair of lines for H2 seasons lay wholly above the pair for B-type seasons, and the excess mortality rate was estimated to be about twice as great for the H2 seasons for each age group. The currently circulating subtypes of A-type influenza (H1, H2, and H3) have circulated in humans only since about the middle of the 19th century [3,4]. These viruses seem to have crossed the species barrier from the avian reservoir, possibly through an intermediate reservoir. A-type viruses mutate (at the nucleotide level) and evolve (at the amino acid level) relatively rapidly. By contrast, B-type influenza viruses have no known animal reservoir, and mutate and evolve at about half the rate of A-type viruses. They seem to be descended from a more ancient ancestor, and are correspondingly better adapted to humans. Possibly as a result, large changes with pandemic effect do not occur with B-type viruses and several lineages co-circulate [5].

Second, in inter-pandemic seasons dominated by A-type viruses, excess mortality generally declined with time, as the population adapted to viruses that had drifted modestly from those of earlier seasons. This occurred significantly more frequently in younger than in older age groups, with the result that the slopes of lines fit to points representing excess mortality in seasons dominated by the same type of virus tended to increase with time. In addition, periods of adaptation were necessarily interrupted by seasons in which mutations produced sufficiently large changes in antigenicity that the immunologic adaptations were abrogated in at least a subset of the population, and re-infection occurred, with corresponding rises in morbidity and excess mortality. The evolutionary dynamics of the A(H1), A(H2) and A(H3) viruses are different [4,6], so the situation became much more complex after 1977 when H1 viruses re-emerged. These have co-circulated since that time along with H3 viruses. H1 viruses have diverse lineages and weaker antigenic drift, but this was not always the case [7]. H1 seasons are currently milder than H3 seasons, producing only slightly higher morbidity and mortality than seasons dominated by B-type viruses.

The largest numbers of deaths associated with influenza infection occurred in the years associated with the emergence of influenza viruses that were novel to most of the world's population, and also in the seasons immediately following this emergence [8]. The phrase 'pandemic age shift' is usually invoked to describe the observed increase in mortality among younger people in the first season of emergence of a novel influenza virus [9]. In the recent 2009 H1N1 pandemic, both cases and deaths exhibited a clear predilection for younger age groups [10-13]. The mini-pandemic of 1977 produced substantial morbidity only in those under the age of 26 [14], and the infamous 'W-shaped' mortality curve of the 1918 pandemic reflected the unusually high mortality rate for 18- to 44-year-olds [15]. This raises several questions: Are younger immune systems more susceptible to and more severely affected by novel viruses? Or is mortality in pandemic seasons merely reset to the virulence level of the emerging virus, except among those immune by virtue of previous exposure, most of whom will be older? Is it possible to distinguish between these hypotheses using the data on pandemic mortality and that in the seasons immediately preceding and following emergence? Increasing our understanding of the epidemiological patterns of mortality during influenza pandemics compared with inter-pandemic periods in different parts of the world could lead to improved influenza control strategies.

In the present study, we generated, analyzed and integrated data on the mortality impact of influenza on the healthcare systems of six economically developed countries in and near the pandemics of the last fifty years of the 20th century.

## Methods

The data for this study were obtained from the national health systems of Australia, Canada, France, Japan, the UK, and the USA [16]. All data available in the 20th century for AC and for pneumonia and influenza (P&I) monthly mortality by age group were used. Wherever possible, 5-year age grouping was used, and all datasets end on 31 December 1999. AC mortality records began on 1 January 1950 for Canada, 1 January 1951 for Japan, 1 January 1959 for the USA and the UK, and 1 January 1968 for Australia and France. The P&I datasets began on 1 January 1950 for Canada, 1 January 1959 for the USA and the UK, 1 January 1968 for Australia and France, and 1 January 1972 for Japan. The age-grouping practice was modified to allow the use of the data on some younger age groups in some of the countries with smaller populations. The Japanese data have not been used previously in any published analysis.

Influenza-attributable mortality rates (deaths per 1,000 of the relevant population or subpopulation) were estimated using a digital filter technique, which produces estimates very highly correlated with those of other methods [17]. This method was selected because it produces estimates that are strictly additive by age group, and are consistent in the sense that they are independent of which seasons are included in the analytical sample (For proofs of these assertions, see Additional file 1. Excel templates for digital filtering of monthly mortality data are available by request from the corresponding author). For each influenza season and country, we fitted a simple exponential model to the estimated excess mortality rate for each available age group [18]. AC excess mortality rates (ACxsMR), were modeled as ACxsMR = *k*1 × exp (*k*2 × age), where *k*1 and *k*2 vary with time and country. The coefficients *k*1 and *k*2 were compared across epidemic seasons and countries by means of 95% confidence intervals (for tabulated results, see Additional file 2).

Mortality rate estimates for the youngest and oldest age groups differed by two orders of magnitude. To enable direct comparisons, we transformed the values of excess mortality for each age group and country using a standard technique, that of centering and scaling C&S). (see Additional file 1 for a description, references, and a table of scaling parameters by country.) C&S standardized excess mortality rates for every age group have a mean of zero and a standard deviation of one. The value associated with each season is the number of standard deviations by which that season's mortality rate differed from the mean for that age group/country. C&S excess mortality rates for all age groups and countries can be plotted on the same graph. We used two graphical forms. For each country, we plotted these rates versus time in a 'rainbow' coloration for age groups, with the oldest age group in dashed black for easy identification (see Additional file 1, Figure S1). We also generated an 'area' form of each rainbow plot in which the area between a peak or trough and zero ordinate was filled with color.

Past pandemics have varied widely in their mortality rates. We sought insight into the two components of age-specific mortality rates attributable to influenza: the attack rate and the case fatality rate. We began with a recent discussion of attack rates for three pandemics [19]. Following the Preferred Reporting Items for Systematic Reviews and Meta-Analyses (PRISMA) statement guidelines (which aims to help authors improve the reporting of systematic reviews and meta-analyses) [20], we used the Web of Science search engine with the following keyword string for the title field: 'influenza' AND '1918', for articles relating to the 1918 pandemic; 'influenza' AND '1957' for articles relating to the 1957 pandemic; 'influenza' AND '1968' for articles relating to the 1968 pandemic; and 'influenza' AND '2009' for articles relating to the 2009 pandemic. We filtered out articles that did not include data on pandemic influenza incidence rates according to age groups. For additional relevant articles, we also examined articles cited in the references of the filtered articles.

## Results

### The A(H2N2) pandemic

**Figure 4:**
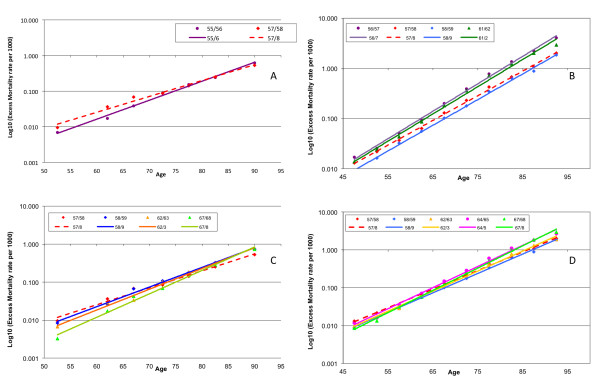
**A semi-log plot of excess All-Cause excess mortality rate variation with age for epidemic seasons during the era of circulation of influenza A(H2N2) viruses in Canada (A) and Japan (B)**. The pandemic season appears as a dashed red line. Old elderly had their lowest mortality in that season. The most recent precedent season dominated by H1 viruses appears in purple. Mortality was generally decreased in the pandemic in Japan. The different time courses of immune escape in these countries are shown in Canada **(C)** and Japan **(D)**

We prepared semi-logarithmic plots of excess AC mortality rates for the seasons before and after the A(H2N2) pandemic in Canada and Japan, respectively (Figures 4A, B), and fitted trend lines to the data for these seasons. *R*^2 ^was greater than 0.99 for all fits to the Japanese data and for all seasons in Canada except the pandemic season 1957/8 and the unusual 1961/2 season. *P*-values for all H2 era seasons for Canada were less than 10^-4 ^(mean 5 × 10^-7^) and for Japan less than 5 × 10^-9 ^(mean 5 × 10^-10^), therefore an exponential relationship provided an excellent fit between AC excess mortality rate and age. The unusually high quality of the fit to the model is also testimony to the consistency of the estimation process, as the digital filter was applied to what should be more or less independent data series.

The excess mortality rate age distribution in the 1955/6 A(H1N1) pandemic season in Canada is shown in Figure 4A (solid purple line). In the subsequent pandemic season, 1957/8, the ACxsMR trend line (red dashed line) had a markedly lower slope (0.1026 ± 0.0146 versus 0.127; *R*^2 ^= 0.985). The pandemic line appeared to rotate clockwise about the age of 80 years with respect to the trend of the 1955/6 season, demonstrating that mortality was lower for groups older than around 80 years, and higher for younger groups. In fact, pandemic mortality for the three highest age groups was lower in the pandemic season than at any time in the subsequent decade (Figure 4C). Relative to the pandemic season, mortality declined for the 1958/9 season (blue line) only for the youngest two age groups; and was sharply higher for all people aged over 65 years, being up to 40% higher for the oldest age group. *R*^2 ^was again greater than 0.99, suggesting that the pure exponential relationship, disrupted in the pandemic, had been reasserted. Throughout the H2 era, the excess mortality rates for the oldest three age groups did not vary by more than 10% and did not show any systematic tendency, whereas younger age groups exhibited serial reductions of more than 40%.

There were several differences in the H2 pandemic in Japan. First, the mortality in the preceding H1 season was generally greater than that in the pandemic. Exponential fits were excellent (*R*^2 ^> 0.99) for every season. The 'axis of rotation' appeared to be about the age of 45 years, that is, mortality was lower in 1957/8 than in 1956/7 for every age group of 45 years and older, and the difference increased with age, from approximately 20% for the youngest of these age groups to approximately 50% for the oldest (Figure 4B). The 1958/9, 1962/3, 1964/5 and 1967/8 H2 seasons were a progression of increases in exponential slope, with each increment in the slope trend being statistically significant (for tables of coefficients and uncertainties, see Additional file 2: XS mortality rates fitted to an exponential model) The reductions in mortality for the youngest age groups were preserved in and after 1964/5, but mortality increased proportionately in all groups aged over 55 years, increasing by over 30% during the 1967/8 season for the oldest elderly group. The 1961/2 season was divergent. We have no information on the dominant virus in circulation for that season in Japan, and so can only note that the age variation is strikingly similar to that in the last H1-dominated season (1956/7), and dissimilar to all other H2 seasons (Figure 4B).

The pandemic reduction in mortality in the old elderly thus lasted only one season in Canada, but extended over seven seasons in Japan. The age variation in excess mortality rate showed no tendency to deviate from a highly significant exponential relationship in Japan in any season, and the time trend to higher slope (that is, of increasing relative mortality risk for the increasingly elderly) was monotonic (Figure 4D).

### The A(H3N2) pandemic in Canada and the USA

**Figure 5:**
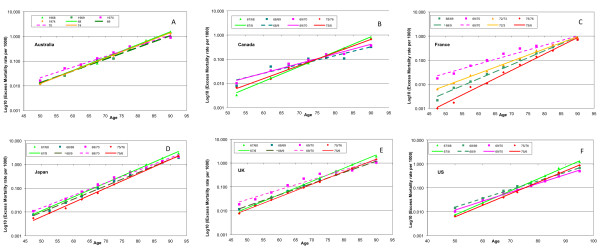
**A semi-log plot of excess all-cause mortality rate variation with age for epidemic seasons before, including and immediately after the emergence of A(H3N2) viruses in (A) Australia, (B) Canada, (C) France, (D) Japan, the (E)UK and the (F) US, in alphabetical order**. The pandemic season is shown as a dashed dark-green line. For countries other than the USA and Canada, the season of emergence was one of low mortality, while the second season was severe (short-dashed fuchsia line). The oldest elderly population over the age of 78 years had reduced mortality in the severe pandemic season, everywhere. This mortality reduction was lost abruptly in the 1975/6 season, except in France.

The emergence of A(H3N2) viruses was very similar in Canada and the USA, but very different in the rest of the world. In the USA, (largest *P*-value, 2 × 10^-7^, mean 7 × 10^-8^) mortality among elderly people was relatively high during the most recent H2 season in the USA, in 1967/8 (Figure 5F, chartreuse line). The pandemic season trend was rotated clockwise from that for the preceding season (dark-green dashed line). The slope change was 0.03 units, and the uncertainty in each estimate was 0.006 units. The 1969/70 seasonal trend was precisely parallel to that for 1968/9, but at two-thirds of the amplitude of the earlier season. The mortality in the next H3 season, 1971/2, was indistinguishable from that of 1969/70; however, in 1975/6, there was a statistically significant increase in slope, a counterclockwise rotation, with younger age groups enjoying a reduction in mortality of about one-third, while the oldest age groups had a 50% increase in mortality rates.

For the emergence of H3 viruses in Canada, the *P*-values for the trends displayed were strikingly higher (mean 4 × 10^-4^), with the highest value, 1.8 × 10^-3^, seen for the pandemic season 1968/9 (Figure 5b). Although this highest *P*-value is still very small, we believe that the relatively low *R*^2 ^value of 0.88 for this particular fit should be taken as corroboration of a visually apparent departure from exponentiality. The trend lines for 1969/70 and 1971/2 were again indistinguishable. The mortality reduction for younger age groups and the 50% increase in the mortality for the oldest elderly groups (those aged over 80 years) in 1975/6 were also similar to those in the USA.

### The A(H3N2) pandemic elsewhere than Canada and the USA

Relative to the most recent season dominated by H2 viruses (chartreuse trend lines), mortality declined in Australia, Japan, and the UK (Figures 5A, D, E) in the pandemic season of emergence (long-dashed dark-green trend lines for 1969, 1968/9 and 1968/9, respectively), but only in the oldest elderly people (aged over 78 years, approximately). The *P*-values for these fits dropped by 10-fold, 25-fold and 2-fold, respectively. For the second season after emergence (1970, 1969/70, 1969/70), the trend line (fuchsia) slope decreased by an amount about equal to the uncertainty in the previous season's estimate, and the *P*-value for the fits dropped another 40-fold, 10-fold, and 10,000-fold (10^-10 ^to 10^-6)^. This is the statistical result of an increase in mortality rates for all ages younger than 75 years, and no change at all for older age groups. Younger age groups experienced progressive reductions in mortality in intervening H3-dominated seasons (1972, 1973/4, 1972/3), but the older age groups had no change until later seasons (1974, 1975/6, 1975/6, when they experienced a 35 to 40% increase in mortality rates. Younger age groups alone exhibited further adaptation (mortality reductions) in 1976 in Australia but not in the other two countries.

The H3 pandemic in France was unusual in a different way (Figure 5C). Unfortunately, age-specific mortality data were not available for the most recent H2-dominated season. In Australia, Japan and the UK, the overall increase in mortality from the first to the second pandemic season for age groups younger than 75 years was 50 to 70%, whereas in France this increase was four-fold (400%; compare dashed dark-green with the fuchsia trend lines), from about half the mortality levels of the three countries listed above to about the level of the highest of these (the UK). There was sequential and proportionate adaptation of younger age groups through the 1975/6 season, but no apparent change in the mortality rates for the oldest age group.

**Figure 6:**
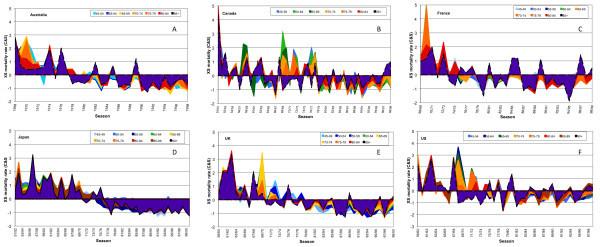
**Area plots of centered and scaled excess all-cause mortality for all age groups for all data available for the second half of the 20th century**. Data are for (A) Australia, (B) Canada, (C) France, (D) Japan, (E) the UK, and (F) the US. Values for the oldest age group are outlined by a black dashed line and filled in purple. Color breakthroughs demarcate seasons of immunoprotection of the old elderly sub-population.

These data are displayed in a different manner for further clarity (Figure 6A-F). Each panel shows the C&S excess AC mortality for one of the six countries using an area coloration scheme, with a dashed black line marking the oldest age group. All peaks after 1967/8 occurred in seasons dominated by H3 viruses. In the USA (Figure 6F), the dashed line traced the top or near-top of every peak except for those for the pandemic of 1968/9 and the following seasonal epidemics, which were dominated by H3 viruses, until the 1975/6 season. The dashed line also outlined nearly all troughs. Beginning in the pandemic season and extending until the 1975/6 season, C&S excess AC mortality for the progressively older population was correspondingly lower, with the oldest age group experiencing mortality below the mean (less than zero C&S). The reduced mortality risk was most dramatically lowered in the progression for the age group aged 75 to 79 years, and we infer that all people of age above the mid-point of that age group (the threshold age) were protected from the pandemic virus and its immediate descendant viruses. A drift variant emerged in 1975/6, the 'Victoria' variant, which abrogated the immunologic protection of these above-threshold elderly people, reinstituting the higher risk of mortality for influenza to the very elderly person. The period 1968/9 to 1974/5 was the only interval in the 40-year data span in which the oldest elderly population was not at the highest relative level of mortality risk.

### The impact of influenza from 1976 to the end of the 20th century

**Figure 7:**
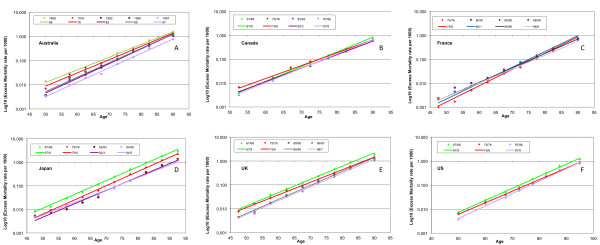
**A semi-log plot of excess all-cause mortality rate variation with age for H3-dominated seasons of immune escape and later**. Six countries ((A) Australia, (B) Canada, (C) France, (D) Japan, (E) the UK, and (F) the USA) are shown, in alphabetical order.

In four countries (Canada, France, the UK, and the USA), there was no change in mortality rates for the oldest three age groups after 1976 (Figure 7B, C, E, F); the lavender to purple-hued trend lines rose to the same point at oldest age for the relevant countries). In Canada, France, and the UK, the trend lines for H3-dominated seasons after 1976 were not statistically significantly different in slope or magnitude from that for the 1975/6 season. In France, there was no apparent trend toward changing slope or magnitude. For Canada and the UK, this possible trend did not reach statistical significance. For the USA, the trend towards progressive adaptation of younger age groups, reflected in increasing slope and decreasing magnitude for seasons after 1975/6 (Figure 7F), became statistically significantly different from 1975/6 only in 1997/8.

In Australia (Figure 7A), the trend lines for the three most severe H3 seasons after 1976 (1982, 1989, and 1997) were significantly different from 1976 in both slope (steeper) and magnitude (lower); however, they were not significantly different from one another. In this case, then, there was a reduction in mortality in all age groups, slightly greater in younger age groups.

In Japan (Figure 7D), the slopes for later H3 seasons (1982/3 and 1994/5 or 1997/8) were significantly different from one another and from the 1975/6 season, but the time trend was toward lower slope, suggesting mortality reduction favoring the older elderly population. Overall, there was a four-fold reduction in the mortality rates for all age groups, about equally between 1975/6 and 1997/8.

### Do pandemic attack rate data provide additional insight into immunoprotection?

**Figure 8:**
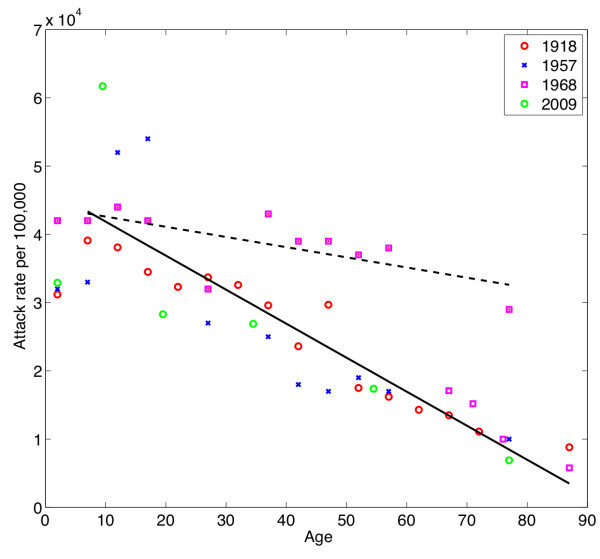
**An estimate of age-specific attack rates hypothetically applicable to all pandemic seasons**. The data displayed are for the pandemics of 1918/9, 1957/8, 1968/9. and 2009/10. The data for 1968/9 come from two studies. The data from Davis *et al. *were fit separately. All other data are fit well by a linear trend that decreased linearly with age.

The entire human population is potentially susceptible to pandemic viruses except for those individuals who are relatively immune as a result of previous exposure to similar viruses in the remote past. If such viruses are so highly transmissible that the general characteristics of social contact among individuals are sufficient to largely determine the age distribution of cases [21] (for example, that each age group has primary social contact with itself), then it is not unreasonable for there to be significant similarities in attack rates across pandemics. The distribution of cases by age in pandemic seasons was well characterized for the 1918/9 A(H1N1) pandemic season in the USA [22] and the 2009 pandemic in the UK [23], but only sparsely for the H2 and H3 pandemics, for which detailed data were available only for a single site for families with school-age children [24] plus a single nursing-home population for the H3 pandemic [25] (Figure 8). There is remarkably little scatter in the data for 1918/9, 1957/8, the nursing-home population from 1968/9, and the 2009 influenza pandemics. However, the data of Davis *et al. *[24] for 1968/9 were essentially flat versus age, and were different not only from those of all other pandemics, but also from the data on older people in a more isolated environment in the same pandemic. Ignoring data for children aged younger than 7 years (for whom the age trend is likely to be positive), the data are fit well by a linear model of negative slope. *R*^2 ^for the fit to all the data except the 1968 data of Davis *et al. *is 0.81 (approximately P = 10^-12^). The data presently do not permit a definitive statement. A pan-pandemic trend, if validated, would greatly simplify future modeling studies. Further study would be valuable because: 1) preparations for every pandemic should then be similar, focused on interrupting contagion, and concentrated on younger age groups; and 2) because pandemic viruses then differ only in their mortality impact (case fatality rates), it becomes interesting to ask whether these differences are qualitative or quantitative.

## Discussion

In this study, we characterized the data for eight pandemic seasons in six economically developed countries: two countries for the A(H2N2) pandemic and all six for the A(H3N2) pandemic. In all these, an older subpopulation of the elderly population experienced reduced mortality in the year of emergence. In countries other than Canada and the USA, mortality was unchanged from the preceding H2 season for all younger age groups in the season of emergence of the H3 viruses, but strongly increased in the second season after emergence, Mortality remained unchanged, however, in the oldest elderly population. It is clear, therefore, that the primary element affecting overall mortality in pandemic emergence is the existence of a relatively immune subpopulation aged above a threshold age. As a result, pandemic mortality was relatively greater in younger age groups in the first season, and absolutely increased over the H2 baseline in the second season, except in Canada and the USA, where these effects could not be separated. We therefore suggest that there is no pandemic 'shift' in mortality to younger age groups, merely a virulence-directed change in overall mortality, modified from a purely exponential variation with age by the relative immunity of persons above a threshold age who had experienced infection in the past with a virus similar to the emerging virus. This mortality sparing was eventually lost in every country. In Australia, Canada (in both the H2 and H3 pandemics), Japan (in the H3 pandemic), the UK, and the USA, mortality increased sharply in the spared age groups in a single season. We call this step up in mortality in previously protected subpopulations 'immune escape,' in epidemiological analogy to the term that was first used to characterize the escape of spontaneously metastasizing tumors from immunological control [26], and was later applied to a discussion of impaired immunological response of T-cells in infectious disease [27]. In France, younger age groups progressively gained mortality reductions until 1975/6, after which time there were no additional gains for any age group. In the H2 pandemic in Japan, all age groups had reduced mortality in the pandemic season, but the protection was proportionately larger with age. This protection was lost sequentially, season by H2 season.

### The A(H2N2) pandemic

The only two countries for which age-specific mortality data were available for both the H3 and H2 pandemics were Canada and Japan. In both countries, pre-pandemic epidemic seasons were well characterized by highly statistically significant, log-linear, positive slope trends with increasing age for excess AC mortality (Figure 4A, B). The pandemic seasons were characterized by overall levels of mortality that were discontinuous with the trends for previous seasonal epidemic years. The pandemic mortality rates were higher or lower than those for previous years, depending on the relative virulence of the emerging virus. In Japan, the H1 season immediately prior to the H2 pandemic was particularly severe. Accordingly, in the pandemic year, every age group experienced a decline in mortality, and this difference increased monotonically with age. Subsequently, post-pandemic mortality rates moved monotonically back toward the trend in pre-pandemic seasons, while maintaining a highly significant exponential relationship

### The A(H3N2) pandemic

The A(H3N2) pandemic was remarkable in that the year of emergence was a season of reduced mortality everywhere in the world except in Canada and the USA, while the second season was severe everywhere. The first season of circulation of A(H3N2) viruses produced severe mortality in Canada and in the USA, taken in its entirety, but mortality was relatively low, not only in most other countries, but also in the Pacific provinces of Canada, the central and mountain regions of the USA, and in Mexico [28]. The basis for this variability is controversial [29,30]. In Japan and the UK, there was a one-year delay (Figure 5D, E), after which the situation played out in essentially the same way as in northern North America. Similarly, in Australia, the first pandemic season was 1969, but severe mortality did not occur until 1970, followed by immune escape in 1974 (Figure 5a). In France (Figure 5c), the first H3 season was also relatively mild; however, the 1969/70 season was extraordinarily severe. Immune escape apparently did not occur in France; rather, there were progressive mortality reductions in younger age groups, with no change in the oldest elderly mortality rates even to the end of the century. This implies that the circulation of H3 viruses was different in France from that in the neighboring UK, possibly in the 1970s, but certainly during the late 19th century when H3-like viruses circulated.

The initial reassortment event that gave rise to the 1968 pandemic A(H3N2) strain apparently resulted in a mismatch between the avian H3 hemagluttinin and the humanized N2 neuraminidase. The H3 viruses that circulated in the late 1800s had equine-like versions of N2 [31,32]. However, the differences in severity in the first H3-dominated seasons in locations other than Canada and the USA probably reflect not only different rates of resolution of functional mismatch of these two gene products [33], but also a sifting of the large number of genomic combinations that were identified in circulation beginning in 1969, owing to an intermixing of the two clades that had emerged in the most recent years of circulation of H2 viruses [34]. The lack of a single course of evolution is especially evident in the schema for the *PB2 *and *PA *genes [4]. In other words, it is the refinement in the field of the performance of a large number of permutations that were generated as H3 replaced H2 in many recombination events, rather than a single genomic substitution, that best explains the sputtering entry of H3 viruses in most parts of the world.

### Comparison of the H2 and H3 pandemics

The mortality patterns of the H2 and H3 pandemics were similar in Canada, with steps up in mortality and a sharp demarcation in age above which immunoprotection was evident. The post-pandemic escape from immunoprotection was also a step in time. The greater mortality attributed to the earlier pandemic was due to the more complete immunoprotection in the H3 pandemic [35]. The pandemic mortality reduction in the H2 pandemic in Japan increased directly with age. The different levels of immunoprotection, recycling ages, and pattern of immune escape in Canada and Japan suggest that there were significant differences between the H2 viruses of the 19th and 20th centuries [36], and that the circulation of these only somewhat similar viruses in the late 1800s produced local variants that were also very different. It may be relevant that Japan was somewhat isolated before its opening to outsiders in the late 1800s.

## Conclusions

### Pandemics do not 'shift' mortality to younger ages

From this study, it is evident that pandemics do not 'shift' mortality to younger ages. Rather, the entire mortality level is simply reset to the virulence level of the emergent virus. This reset is accompanied by immunoprotection in older age groups, which is determined by their level of previous experience with viruses similar to that emerging.

### All known pandemics have involved 'recycled' viruses

It is well accepted that older persons (aged over 45 to 55 years of age) were spared in the 1918 A(H1N1) pandemic [37,38], although as reported above for H2 viruses, recent data from central Mexico and Colombia suggest geographic differences in pre-existing immunity to these viruses [39,40]. We and others have shown that older people (aged over 62 years), were spared in the 2009 H1N1 pandemic [10] and individuals older than 26 years were reported to be spared in the mini-pandemic re-emergence of H1N1 viruses in 1977/8 [14]. Therefore, it appears that in all five events over the past 100 years in which an influenza virus emerged with pandemic effect, a significant proportion of then-living individuals aged above a particular threshold age were immunologically protected from the emergent virus. Such viruses are said to be 'recycled' [25]. Our data are consistent with sero-archeology studies in which antibodies to pandemic viruses have been identified in age-cohort sera drawn before the H3 pandemic [41]. Unfortunately, the sero-archeology data on past circulation of H2 viruses is inconsistent. As we noted above, the 2009 pandemic virus was most similar to H1 viruses that circulated before 1947. Therefore, the concept of recycling must be broadened to include not only pandemic viruses, but also antigenically significant variants of emergent viruses. Lessler *et al. *[42] have recently shown that sero-archeology curves (plots of antibody levels in age cohorts versus the cohort age) of pandemic viruses and their variants are essentially indistinguishable, except for a displacement in time to the date of emergence of the variant.

### Immunoprotection from recycled viruses is a cardinal attribute of pandemics

It is clear that immunoprotection from recycled viruses is a cardinal attribute of pandemics, and this conclusion leads logically to several corollaries, as follows.

#### Immunoprotection should direct pandemic planning

Vaccines will not be required for protection for those aged above the age of recycling (Above the Age of Recycling Persons; AARPs), for both pandemic seasons and epidemic seasons dominated thereafter by variants of the pandemic virus, until immune escape occurs. Therefore, this otherwise high-risk population segment, easily defined in advance for any candidate pandemic virus, can be provisionally removed from pandemic vaccine planning. As there is a consensus among those modeling vaccine performance that deploying vaccine first to children and working adults should provide more effective protection, and as global vaccine production is inadequate, this should improve the level of effective coverage. Of course, if an AARP should become infected with pandemic influenza, the high *a priori *expected case fatality rate implies that antiviral resources should be made immediately available to them.

#### Immunoprotection does not prevent infection, only the consequences of infection

We have remarked that the data on variation in pandemic attack rate with age appears surprisingly similar across all four pandemics, suggesting a linear decline with age. We found similar threshold ages above which immunoprotection from mortality was present in both the H2 and H3 pandemics. However, these thresholds are both higher than the age threshold for protection in the 2009 H1 pandemic (approximately 62 years), the 1918 H1 pandemic (45 to 55 years) and the 1977 mini-pandemic (approximately 26 years). If a universal attack rate for pandemics is a viable hypothesis, then this must mean that immunoprotection from mortality does not influence the infection rate, but only the severity of its consequences. It could even be argued that a universal pandemic attack rate is the epidemiologic correlate of the well-known immunologic observation that the control of the spread of virus infections lies in the rapid response of memory B- and T-cells, but that circulating levels of antibodies and armed T-cells are insufficient to prevent viral entry entirely [43,44].

#### Immunoprotection must be explicitly considered in vaccine trials

Trials of vaccines and studies that analyze the effects of influenza vaccination should either exclude or analyze as positive controls the already immune AARP population segment, both for the pandemic season and for the following seasons until immune escape occurs. After immune escape occurs, and until a future time when descendant viruses have mutated so completely that infection no longer stimulates the pattern of response conditioned by exposure at an early age to similar viruses (that is, original antigenic sin, OAS [45]), AARPs will falsely appear to be vaccine failures. The degree of vaccine effectiveness will necessarily be very low, possibly even negative in the first season of immune escape, and will rise as the evolutionary distance from the pandemic virus increases [46]. Unfortunately, little is known about the restoration to immunological grace after OAS.

#### Immunoprotection may be a key to pandemic survival for organizations too

If pandemic attack rates are indeed universally low for the pre-immune elderly person, employers should consider prospectively recruiting AARPs for positions crucial to the missions of their enterprises. AARPs experience seasons of emergence as no more severe than seasonal influenza epidemics. Absenteeism among working-age populations in severe pandemics could reach levels of 50% or more. Assembly line-like activities can usually tolerate no more than 20% absenteeism. It is true that the mortality rate of AARPs is likely to be absolutely higher than that of the younger population in pandemics; but it will be relatively lower, and the attack rate for AARP's will be absolutely lower.

#### Immune escape may be accompanied by substantial increases in mortality and morbidity

Our data show that immune escape may be immediate or delayed for several seasons. This may also be seen in reports analyzing the 1918 pandemic, from which immune escape, as an increase in excess mortality among the elderly population, did not occur until 1926 [47,48]. There is some genomic insight into the evolution of H3 strains, A series of amino acid changes occurred between 1971 and 1975 [49]. Coincident with the loss of immunoprotection in AARPs in 1975/6, the variant A/Victoria/3/75 appeared, in which two glycosylation sites were added onto the globular head of HA, at positions on HA 81 and 126, while losing a site at position 63. These nascent glycosylation sites were located within antigenic sites E and A, respectively, and the site that was lost at position 63 was also within antigenic site E. We hypothesize that this change in glycosylation, particularly the addition of the position 126 site within antigenic site A, abrogated the immunoprotection of the OAS response in AARPs, allowing re-infection [50]. Lessler *et al. *[42] found that H3/Victoria/3/75 was the least antigenic of the nine H3 viruses in their study, all of which circulated sequentially. This is surprising given the large excess mortality rate estimated for the 1975/6 season. Two studies have reported that vaccination of humans with inactivated viruses does not produce an OAS effect, that is, induced antibody shows a higher affinity to a previously encountered influenza virus strain than to the virus strain present in the vaccine [51,52]. However, it may be of significance that the OAS strain in the first of these studies was Victoria/3/75. Kim *et al. *[53] found that sequential administration of inactivated virus vaccine resulted in a higher viral load and defective clearance in mice on challenge with the second virus in the sequence, and they also found (again in mice) a large OAS effect on serial natural infection, independent of order. Possibly more importantly, Zhou and Deems [54] have argued that, in dengue (for which there are four known viral subtypes), OAS is augmented when infection with a highly antigenic subtype is followed by infection with a cross-reactive subtype that is significantly sub-dominant to the first, and is muted in the reverse situation (first infection by a sub-dominant subtype followed by second infection with a more dominant subtype). This would appear to be the situation that existed in 1975/6 with the emergence of the Victoria/3/75 variant. Zhou and Deems provide a mathematical model of T-cell response that seems to account generally for the observed response. If an analogy may indeed be extended to influenza, managing the T-cell response is an important feature currently lacking in influenza vaccines that needs to be addressed if immune escape is to be mitigated.

If an emergent virus is, like novel H1N1 of 2009, a virus recycled from a point well along in its adaptation to the human species, antigenic sites will have drifted in both amino acid sequence and in the generation of glycosylation sites. Because the 2009 virus is relatively non-virulent, the analogy with dengue would suggest that the potential for an augmented OAS response accompanying immune escape for this virus is relatively low. SOIV 2009 offers a rich opportunity for the study of immune escape, because many of the mutation pathways potentially open to this virus are precluded by the continued circulation of H1N1 viruses that re-emerged in 1977. With a relatively low recycling age (> 62 years) the number of susceptible people, initially low, is now further reduced. Evolutionary pressure on this virus must be enormous, thus rapid change or extinction should be anticipated.

#### The mortality rate attributable to influenza was diminished after 1976, but not as a result of vaccination

Over the second half of the 20th century, Canada and the USA had, by far, the lowest influenza-mortality rate, about half that of the highest rate in these countries, which was in the UK. However, a common feature of our graphs (Figure 6; see Additional file 1, Figure S1) is that after 1975/6, only a few mortality peaks anywhere exceeded the mean excess mortality (C&S = 0). Compared with the mean excess mortality in the 25 years before 1980, excess mortality for the period 1980 to 2000 was 40% lower in Japan, about 20% lower in Australia and the UK, 10% lower in France, and 5% lower in Canada and the USA. Both mortality and variation came to be highest in the UK, among these six countries. There was no discernible trend in influenza-related mortality in four of the six study countries after 1976 (Canada, France, the UK. and the USA). In Australia, mortality rate models of H3 seasons after 1976 differed significantly, showing mortality reduction in all age groups, but proportionately less in the older elderly population. Only in Japan, where curiously, the elderly population was not vaccinated at all, was there evidence of mortality reduction favoring the older elderly population. For the peak mortality years (all H3 seasons), the oldest elderly population in all countries had the highest relative mortality (Figure 6, dashed lines). By contrast, in type B and A(H1N1)-dominated seasons (the troughs), the oldest elderly population tended to have either the lowest relative mortality, or had values that were little different from those of younger people. These observations are remarkable because programs for influenza vaccination principally targeting the elderly population were instituted in all of these countries except for Japan, and these programs dramatically increased in coverage after 1980. In Japan, influenza vaccination targeted only children in a program that was begun in 1972 and that terminated in 1987 [55]. None of the elderly population received any vaccine, and no influenza vaccine was deployed in Japan during the period 1993 to 1996/97, after which a more conventional vaccination program including elderly people was deployed.

Could influenza vaccine be more effective in B-dominated and H1N1-dominated seasons than in those dominated by H3 viruses? This would run counter to the conventional wisdom that inactivated influenza vaccines induce lower rates of seroprotection against B viruses than against either H3N2 or H1N1 viruses [56]. The lethality of H3 viruses and the benign nature of B-type and H1N1 viruses for the elderly person is possibly better explained by the relatively low mutation rate of B-type influenza viruses and greater historical experience with viruses similar to the H1 viruses. In any case, it is clear that the pattern of influenza-attributable mortality, including the overall decline, has been unaffected, at the population level, by the distribution of influenza vaccine.

If there is no evidence that excess mortality has declined in the older elderly population, who have been subject to the greatest increase in and the most comprehensive coverage with influenza vaccination; if the only decrease in excess mortality among the older elderly occurred in the one country that did not vaccinate its elderly population; and if an argument can be made that conventional, inactivated virus vaccines may even engender a defect in viral clearance, then the trial design for vaccines should be re-examined. One of several potential issues these observations raise is the use of placebo controls. The well-characterized lack of efficacy in the elderly population [57-59] suggests that ethical concerns about placebo use may be misplaced. Detection of vaccine effects is intrinsically difficult. Detection of such effects in a population with little expected effect requires large samples and control of all anticipated confounders. We suggest that investigation of influenza vaccines in elderly populations might be best performed using as many such controls as possible. Additionally, if recent progress in understanding the immunology of the dengue virus has a valid analogy or indeed any application to influenza, then the immune escape of AARPs is potentially fraught with particular peril, because pandemic viruses are often highly immunodominant, whereas subsequent variants are less so. It is likely that new forms of influenza vaccine [60] and possibly new modes of administration may be needed to protect those elderly people who were initially immune, from the effects of exposure to variants of emergent viruses at and after immune escape.

## Abbreviations

AARP: Above the age of recycling persons; AC: All-cause (used for both mortality due to all causes and excess mortality due to all causes); ACxsMR: All-cause excess mortality rate; B-type: Influenza subtype B; C&S: Centering and scaling; H1, H1N1: Influenza subtype A(H1N1); H2, H2N2: influenza subtype A(H2N2); H3, H3N2: Influenza subtype A(H3N2); N2: Influenza type A (neuraminidase type 2); OAS: Original antigenic sin.

## Authors' contributions

TR acquired the data, performed excess mortality calculations, prepared draft plots of Figures 1, 2, 3, 4, 5, 6, 7 and Additional file 1, Table S1, and drafted the manuscript. GC provided key references and insight, developed the universal attack rate model, performed the analysis of the exponential model of excess mortality, generated drafts and revisions of Figure 8, and prepared final versions of all figures. JAM provided continuous feedback, was chiefly responsible for the integration of molecular genetic and genomic data, and identified viral evolutionary events likely to be responsible for immune escape. All three authors contributed to the design and form of the manuscript, reviewed and commented upon all drafts, and have read and approved the manuscript for publication.

## Competing interests

TR reports receiving about $5,000 in 2010 for services provided to Novartis, lnc. in the review of influenza vaccine clinical trials. The material of this paper was developed subsequent to his work with Novartis. Neither Novartis nor any other body exerted any influence upon the authors with regard to the data or the interpretation thereof used in this paper, and no one except the authors and Drs D.S. Fedson, W.P. Glezen, and A-J Valleron (esteemed colleagues and former co-authors) have reviewed the contents. Apart from the above, there are no potential conflicts of interest known to any of the authors.

## Pre-publication history

The pre-publication history for this paper can be accessed here:

http://www.biomedcentral.com/1741-7015/10/162/prepub

## Supplementary Material

Additional file 1 **Exposition of Methods and Models**. Details of techniques used are presented, together with formal proofs of the claimed properties of the method for estimating excess mortality. This file also includes a rainbow coloration version of the text Figure 6 to permit the reader to estimate the actual data used. Click here for file

Additional file 2 **XS mortality rates fitted to an exponential model**. This Microsoft Excel file contains estimates for the coefficients of an exponential fit to every season for which data were available for each of the six countries studied. Click here for file
