# Supplementary material for: The age distribution of mortality due to influenza: pandemic and peri-pandemic
Source: BMC Med. 2012 Dec 12;10:162. doi: 10.1186/1741-7015-10-162 (PMC3554498; doi:10.1186/1741-7015-10-162)
Supplement: Additional file 1 — Exposition of Methods and Models. Details of techniques used are presented, together with formal proofs of the claimed properties of the method for estimating excess mortality. This file also includes a rainbow coloration version of the text Figure 6 to permit the reader to estimate the actual data used. [file 1741-7015-10-162-S1.DOC]

**Additional File 1: Exposition of Methods and Models**

**Methods and Data used**

The data for this study were obtained from the national health system of Australia, Canada, France, Japan, the UK, and the US [1]. All data available in the twentieth century as monthly mortality by age group, attributable to all-causes (A-C) and to pneumonia & influenza (P&I) were used. Wherever possible, 5-yr age groupings were used. All datasets end at December 31, 1999. A-C mortality records begin 1/1/50 for Canada, 1/1/51 for Japan, 1/1/59 for US & UK, and 1/1/68 for Australia and France. P&I datasets began on 1/1/50 for Canada, 1/1/59 for US & UK, 1/1/68 for Australia and France and 1/1/72 for Japan. The age grouping practice was modified to allow the use of some younger age groups in several countries with smaller populations.

Mathematical estimates of excess mortality are based on the assumption that mortality rises in winter and precipitously so during the periods in which influenza is epidemic. Influenza-attributable mortality is necessarily an inferred quantity since most deaths actually due to influenza are recorded as deaths associated with other causes. We used a technique for estimating excess deaths that was previously demonstrated to isolate highly similar information from mortality series associated with each of the different disease conditions that display a winter increase in the US. [2] This technique operates on interval mortality corrected to a standard time interval [[1]](#footnote-2) and uses a digital filter to extract long-term trends and produces estimates for influenza-attributable mortality highly correlated with other methods. It has the additional properties that the estimates for any season do not depend on which other seasons are included in the analytical sample; and in the configuration used here, that the estimates for all age groups are strictly additive. This is important because we make comparisons across countries and age groups. Below we include a summary of the technique and proofs of the special properties. The reader should be comfortable that the estimation of excess mortality is a mature technology, that all methods yield highly correlated values, and that results here utilize values scaled in a standard fashion.

The Digital Filter Method of estimating excess mortality

This material provides a description of the digital filter methodology used to estimate excess mortality in the accompanying paper, and includes a demonstration that the estimates provided by this method are independent of which other seasons are included in the analytical sample and are strictly additive. Without the first property, the estimates for any particular season will vary depending on what other seasons are included in the analytical sample. Serfling and regression models, including poisson regression, do not have this property, which is important for the work of this paper because the analytical samples of mortality attributable to various disease classes and age groups span different ranges of seasons both within and across countries. Adding or subtracting seasons will not change our results. A proof of this appears in the pentultimate section of this Supplementary Material

Additivity is important in this study because we are directly comparing estimates for different age groups both within and across seasons. Since regression methods are not constrained to produce additive results, i.e., the regression is performed for each age group separately, those estimates are not additive. A proof of the strict additivity of the digital filter technique and the resultant constraint on its use is given as the last section of this Supplementary Material,.

The digital filter employed in this work is a 13-month moving average modified by a window to avoid artifacts. It is simple to employ; and requires only 3 parameters which characterize the length of the averaging and the characteristics of the window. These are independent of the data. The digital filter produces results similar to those of more complicated methods for estimating excess mortality [3, 4, 5], for very little effort, with better properties. In particular, this method can gain the property of additivity of age groups by using the same set of months as the influenza period for every age group. The proof is straightforward, and is presented here in the last section. It is not clear, however, that requiring summation over the same set of months for each age group is a good representation of nature. This constraint neglects the observations that children, adults and seniors have very different periods of infectivity in most epidemics, with young children leading in presentation by several weeks, followed by young adults and well lagged by seniors [cf. 6].

It is usual for analysts to utilize external information to determine the time period associated with an influenza epidemic. Many authors continue to use the time period in which Pneumonia & Influenza-specific mortality (P&I) is clearly elevated above an estimated background as the defining point for influenza epidemics [cf. 3]*.* This practice seemed unwise with the data of this study, because the fraction of excess All-Cause (A-C) mortality attributable to P&I, while nearly 100% in the work of Collins in the 1918-1935 timeframe, fell to about 40% in the decade 1937-1947 and since 1968/9 has been below 20% (range: 7-18%) in all countries considered in this study, except the UK where the average was 24%. Inspection of the data easily demonstrated that P&I mortality rises before and declines variably with respect to All-Cause mortality. Since it is the properties of All-Cause mortality upon which we are focused, we have used the epidemic period evident in the estimation of excess mortality for the age group GE 65 years to define the influenza epidemic period for the All-Cause estimates and the P&I estimates, separately.

It is important to note that in all of these countries, for A-C mortality, age groups from age 20 to age 45 do not have winter peaks in mortality, but rather have summer peaks and winter troughs because the dominant cause of death in this age range was accidents. Estimation could still be performed by subtracting from all cause mortality all causes of death which, separately, do not exhibit a winter peak. Because our focus was on the older half of the population, we have not introduced this additional complexity.

Microsoft Office Excel templates for calculating the excess mortality from a column vector of mortality attributable to all-causes or any disease, specific to any age-group can be obtained directly from the corresponding author.

The Technique of Centering and Scaling (C&S)

This operation is also known as the standard score or Z-score transformation. The average of excess A-C mortality for each age group within a country was subtracted from each seasonal estimate of excess mortality for that age group, and the difference was divided by the standard deviation for that age group and country. Therefore, zero C&S is the average excess mortality rate for every age group [cf. 7]. An alternative description for the result of the C&S operation is the deviation from the mean expressed in units of the standard deviation.

Table S1 summarizes the parameters used for scaling the excess all-cause mortality for the age group, > 64 years, for the full range of available data in the six countries studied and for the period 1980-1999.

| **Country** | **Average excess All-cause**  **mortality rate – age Ge 65** | | **Standard**  **Deviation** | |
| --- | --- | --- | --- | --- |
|  | All dates available | 1980-99 | All dates | 1980-99 |
| Australia | 176.5 | 145.8 | 59.4 | 36.0 |
| Canada | 108.8 | 104.1 | 35.9 | 30.7 |
| France | 134.6 | 120.6 | 48.7 | 34.4 |
| Japan | 216.9 | 126.3 | 118.9 | 23.7 |
| UK | 240.7 | 193.4 | 96.2 | 55.3 |
| US | 112.3 | 109.1 | 26.5 | 22.3 |

Table S1:Computed parameters used in Center & Scale transformation for the age group, > 64 years for all data available in six countries for the time period, 1980-1999

Models of the exponential variation of influenza-attributable mortality with age

For each influenza season and country, we fit a simple exponential model to the estimated excess mortality for each available age group. All-cause excess mortality rates, A-CxsMR, are given by A-CxsMR(age)= k1 exp ( k2 * age), where k1 and k2 vary with time and country. The results are tabulated below for reference.

Tables of k1, k2, R2, P, and uncertainties on the coefficients are provided in Additional File 2 - XS mortality rates fitted to an exponential model, an Excel file

Graphical Display of Excess All-Cause Mortality for all age Groups and Countries for the last half of the last century

The graphs (Figure S1a-f) that follow below use a consistent coloration (rainbow plots) by age group to display the time variation of excess all-cause mortality for the six economically developed countries in the study. The objective is to provide readers with a more detailed interest in this topic with access to estimates of the values. Some of the researchers in this study preferred this display to the area plots used in the manuscript. It is, for example, much easier to see in this display form that, in the US, the age group, 75-79 years, is split in its immunoprotection.

Excess mortality, as estimated for any season by the digital filter method, is independent of which other seasons are included in the analytical sample

In the digital filter methodology, excess mortality for a disease class, r and an age group, k, is estimated as the difference between the elements of an observed mortality time series, s[r,k]_m_, and the isochronous elements of a baseline series, s[r,k]_b_, summed over a particular set of time intervals, called the epidemic periods. The baseline series used in the digital filter method is the series generated by a weighted central moving average of 13 points centered around each mortality series element. A moving average of 13 points was chosen because the mortality data for this study were monthly interval data and the filtering objective was to include in the baseline effects with a frequency of annually and longer. To avoid filtering artifacts due to the finite length of the moving average, the elements of the moving average are weighted using a Hamming window [8]. Hamming window coefficients or weights, w_n_ , are given by

w_n_ = 0.54 – 0.46 cos(2π*n*/(*N* – 1)), 0 ≤ *n* ≤ (*N* – 1), where *N* = 13 in our usage. We obtained the values 0.080, 0.142, 0.310, 0.540, 0.770, 0.938, 1.000, 0.938, 0.770, 0.540, 0.310, 0.142, and 0.080.

We have the following definitions:

N N-6 i+6 N-6

s[r,k]_m_ = {m[r,k]_i_} ; s[r,k]_b_ = {b[r,k]_i_} = { (1/6.56) Σ w_j-i+6+1_ m[r,k]_j_ } ; and

i = 1 i =7 j = i-6 i = 7

N-6 N-6

s[r,k]_d_ = {d[r,k]} = {m[r,k]_i_ –b[r,k]_i_ }

i = 7 i = 7

The excess mortality (XSM) estimated for any age group, k, and season, o, is given by the expression,

q_o_

XSM[r,k,o] = Σ d[r,k]_i_ ,

i = p_o_

where p_o_ and q_o_ are the first and last elements in the difference series, s[P&I or A-C, ³ 65]_d_ , the mortality series for deaths due to pneumonia and influenza (P&I) or all causes (A-C) and the age group, ³ 65 years, for which values of d[P&I or A-C,³65] are contiguously positive during the o-th winter season. Since all the elements of sum in XSM[r,k,o] are weighted or unweighted elements of the mortality series, {m[r,k]} for the years o-1, o and o+1, the value of XSM[r,k,o] depends only on mortality values for these years, and is unchanged by the presence or absence of other years and seasons in the analytical sample.

Excess mortality estimated by the digital filter method is strictly additive by age group.

The observed mortality for a group of individuals is the sum of the mortalities of each constituent age group, e.g., for an age group, k, comprised entirely of individuals of age groups, a1 and a2, each observed mortality,

m[r,k]_i_ = m[r,a1]_i_ + m[r,a2]_i_ ; b[r,k]_i_ = b[r,a1]_i_ + b[r,a2]_i_ ; and d[r,k]_i_ = d[r,a1]_i_ + d[r,a2]_i_

It follows then that

q_o_ q_o_

XSM[r,k,o] = Σ d[r,k]_i_ = Σ d[r,a1]_i_ + d[r,a2]_i_ = XSM[r,a1,o] + XSM[r,a2,o]

i = p_o_ i = p_o_

Since a1 and a2 are arbitrary age groups, and since the argument holds for any number of constituent age groups, it follows that excess mortality as estimated by the digital filter is strictly additive by age group.

**Figure Legends**

Additional file 1, Figure S1 a-f. Rainbow-coloration plots of centered & scaled excess All-Cause mortality for all age groups of all data available for the last half century are provided to allow the interested reader access to data detail. Data are for Australia (S1a), Canada (S1b), France (S1c), Japan (S1d), the UK (S1e), and the US (S1f). Values for the oldest age group are outlined in a black dashed line.

**
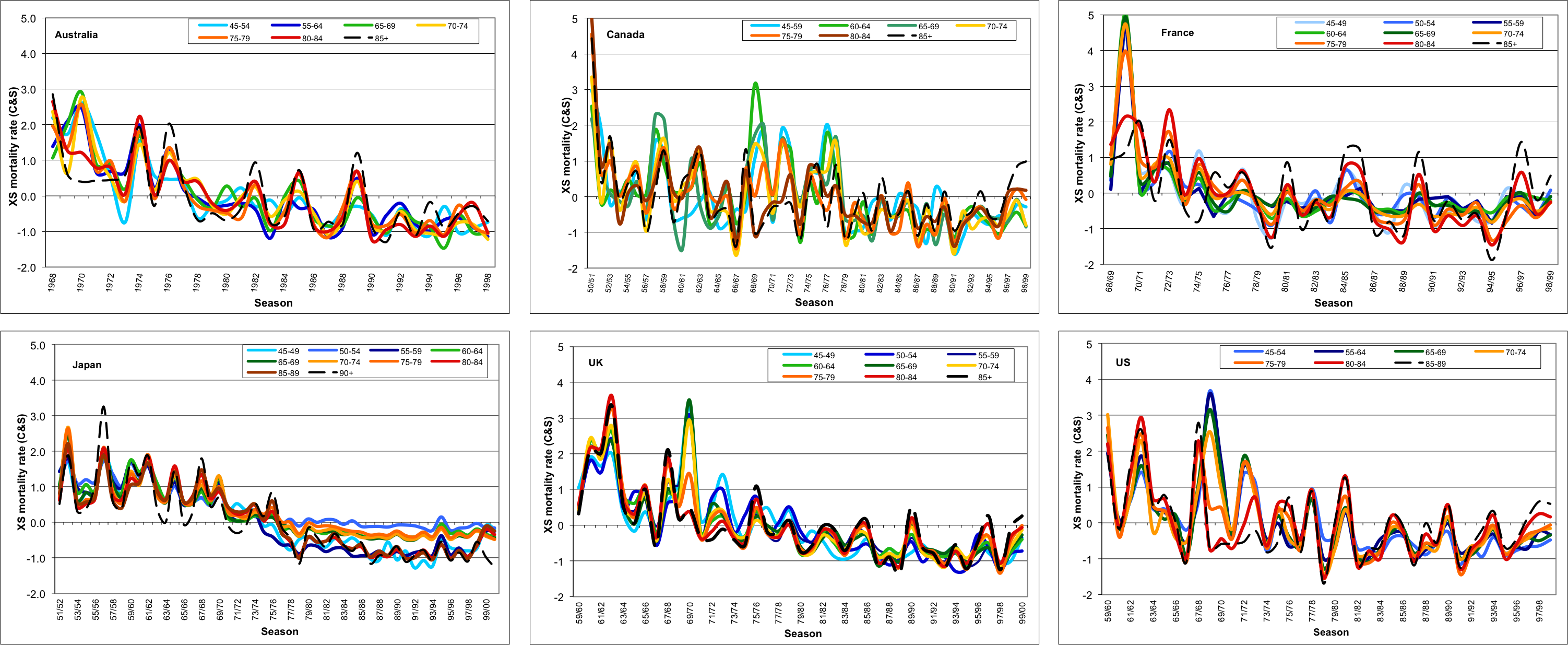
**

**Figure S1.**

**References**

1 Australia: ABS Cause of Death, Australia (cat 3303.0) Unpublished data available on request.

Canada: Special tabulation, Canadian mortality database, Statistics Canada, Ottawa, Ontario, (2003).

France: Data from the “Centre d‘epidemiologie sur les causes medicales de deces”, <http://www.cepidc.vesinet.inserm.fr/cgi-bin/broker.exe>.

Japan : Vital Statistics of Japan: Deaths by sex and age, month of occurrence and year of birth. These paper files were provided by Dr M Onishi of Becton Dickinson & Co and were extracted for electronic usage by Applied MicroImage Corporation, Waltham, MA.

UK : All-cause, disease specific monthly mortality and population data were provided by Dr Alex Elliot of the Birmingham Research Unit of the Royal College of General Practitioners

US : National Center for Health Statistics. Vital Statistics of the United States, “Mortality”, public use files, 1959**-**1999.

2 Reichert TA, Simonsen L, Sharma A, Pardo SA, Fedson DF, Miller MA**. Influenza and the winter increase in mortality in the United States, 1959-99.** *Am J Epidemio*l 2004, **160**: 492-502

3 Thompson WW, Shay DK, Weintraub E, Brammer L, Cox N, Anderson LJ, Fukuda K. **Mortality associated with influenza and respiratory syncytial virus in the United States*.*** *JAMA* 2003; **289:** 179-186.

4 Tillett HE, Smith JW, Gooch CD. **Excess deaths attributable to influenza in England and Wales*.*** *Int J Epidemiol* 1983; **12:** 343-52.

5 Saglanmak NS, Andreasen V, Simonsen L, Molbak K, Miller MA, Viboud C. **Gradual changes in the age distribution of excess deaths in the years following the 1918 influenza pandemic in Copenhagen: Using epidemiological evidence to detect antigenic drift**. *Vaccine* 2011; **29S**: B42-8.

6 Brownstein JS, Kleinman KP, Mandl KD**, Identifying Pediatric Age Groups for Influenza Vaccination Using a Real-Time Regional Surveillance System***. Am J Epidemiol* 2005; **162**: 686-93.

7 Stockburger DW. Introduction to Statistics: Concepts, Models and Applications, Chapter on Linear Transformations: <http://www.psychstat.missouristate.edu/introbook/sbk15.htm>

for more detail and insight.

8 Oppenheim AV, Schafer RW. *Digital signal processing*. Englewood Cliffs, NJ, Prentice-Hall, 1975, ed. 1: 239–50.

1. In this case, monthly mortality and a standard 30.41 d month [↑](#footnote-ref-2)
